# Improvement of Foaming Ability of Surfactant Solutions by Water-Soluble Polymers: Experiment and Molecular Dynamics Simulation

**DOI:** 10.3390/polym12030571

**Published:** 2020-03-04

**Authors:** Chaohang Xu, Hetang Wang, Deming Wang, Xiaolong Zhu, Yunfei Zhu, Xing Bai, Quanlin Yang

**Affiliations:** 1 School of Safety Science and Emergency Management, Wuhan University of Technology, Wuhan 430070, China; xchwhut@whut.edu.cn; 2 Key Laboratory of Gas and Fire Control for Coal Mines (China University of Mining and Technology), Ministry of Education, Xuzhou 221116, China; dmwang@cumt.edu.cn (D.W.); zyfspirit@foxmail.com (Y.Z.); 3 State Key Laboratory of Fire Science, University of Science and Technology of China, Hefei 230026, China; cqrczxl@163.com; 4 School of Urban and Environment, Yunnan University of Finance and Economics, Kunming 650221, China; baixing1994@163.com; 5 Academic Journal Center, Xi’an University of Science and Technology, Xi’an 710054, China; quanlinyang@xust.edu.cn

**Keywords:** surfactant, water-soluble polymer, foaming ability, molecular dynamics simulation

## Abstract

Aqueous foam is widely used in fire extinguishing and dust suppression technologies. Improving the foaming ability is the key to reducing the added concentration of foaming agents as well as the economic cost. In this work, the effect of a water-soluble polymer (polyvinyl alcohol, PVA) on the foaming ability of anionic surfactant (sodium dodecyl ether sulfate, SDES) was studied by an experiment and molecular dynamics simulation. The experimental results showed that PVA greatly improves the foaming ability of SDES solutions when the surfactant concentration is less than 0.1%, which is attributed to the fact that the polymer can enhance the stability of bubble films and reduce the bubble rupture rate during the foam generation process. The simulation results indicate that PVA can enhance the hydration of surfactant head groups and contribute to the formation of a three-dimensional hydrogen bond network between surfactants, polymers, and water molecules, thus greatly improving the stability of bubble liquid films. The above results suggest that water-soluble polymers can be used to improve the foaming ability of surfactant solutions by enhancing the bubble film stability, which is beneficial as it reduces the added concentration of foaming agents in aqueous foam applications.

## 1. Introduction

Coal plays a vital role in worldwide energy consumption and the supply of chemical raw materials [1,2,3,4]. Statistics indicate that coal accounts for 27.2% of primary energy consumption. The world’s major coal producers include China, the United States, India, Indonesia, Australia, and Russia. Among them, China accounts for 46.7% of the global coal production [5]. Many serious disasters occur in the coal mining process [6,7,8,9,10], such as fires and dust accidents [11,12,13,14,15]. Coal fires not only burn away a great quantity of nonrenewable resources but also release toxic gases, causing asphyxiation and air pollution [16,17]. Additionally, large amounts of dust are generated when cutting coal and rock, which can trigger coal dust explosions and pneumoconiosis [18,19,20,21]. In order to create a safe working environment for coal miners, aqueous foam has been proven to be very effective for extinguishing fires and suppressing dust; these products are known as foam extinguishing technology and foam dust suppression technology [22,23,24,25,26]. In field applications, foam is produced by injecting compressed air into foaming agent solutions [27]. The problem is that the added concentration of foaming agents is relatively high. For example, the concentration of foaming agents for firefighting is 0.8%–3% [22,28,29], and the foaming agents for dust suppression are used at a concentration of 0.5%–3% [30,31]. The high economic cost severely restricts the widespread application of foam extinguishing technology and foam dust suppression technology. The only approach used to lower the concentration is to improve the foaming ability of foaming agents, so that sufficient foam can be generated at low concentrations, thereby reducing the economic cost.

Surfactants are the main component of foaming agents. To improve the foaming ability of foaming agents, some scholars have tried to add water-soluble polymers to surfactant solutions [32]. Xu et al. [33] reported that the water-soluble polymer welan gum increased the foaming ability and foam stability of the anionic surfactant sodium fatty alcohol polyoxyethylene ether sulfate (AES) solution. Deng et al. [34] investigated the influence of the water-soluble polymer exopolysaccharide (EPS) on the foam properties of anionic surfactant AES solutions and found that both the foaming ability and foam stability of the AES/EPS solutions were considerably higher than those of single AES solutions. Zhang et al. [35] studied the effect of bovine serum albumin (BSA) on the foaming ability and foam stability of Tween 20 solutions. They discovered that BSA reduced the foaming ability of surfactant solutions but increased the foam stability. The effect of the water-soluble polymer partially hydrolyzed polyacrylamide (HPAM) on the foaming ability or the foam stability of different surfactant solutions was reported by Ahmed et al., Azdarpour et al., and Xu et al. [36,37,38]. Experimental results showed that HPAM could improve the foaming ability and foam stability of surfactant solutions. Chen et al. [39] evaluated the foam stability of α-olefin sulfonate (AOS) solutions after adding HPAM or xanthan gum (XG). It was observed that the foam stability of surfactant-polymer complexes was higher than that of single surfactant solutions. Wang et al. [40] added HPAM or XG into the ionic surfactant sodium dodecyl benzene sulfonate (SDBS) solution and tested the foaming ability, liquid holdup, and foam appearance. Relevant results suggested that the foaming ability of SDBS solutions was enhanced by the addition of polymers, and the efficiency of XG was higher than that of HPAM. Wang et al. [41] illustrated that the polymers HPAM, hydroxyethyl cellulose (HEC), and polyvinyl alcohol (PVA) greatly increased the foam stability of two kinds of surfactant solutions, but they did not investigate the foaming ability. Although many water-soluble polymers have been studied in relation to the foam properties of surfactants, very little attention has been devoted to the effect of PVA on the foaming ability of surfactant solutions, and the mechanism of foaming ability improvement by PVA is still not clear.

Exploring the interaction between surfactants and PVA at the microscopic level could help to explain the mechanism by which polymers improve the foaming ability of surfactant solutions. With the rapid growth of computational power, molecular dynamics simulations have become an effective tool for studying the adsorption behaviors of surfactants and polymers at the air–water interface. For instance, Mafi et al. [42] investigated the complex formation between surfactants and HPAM on the water surface using molecular dynamics simulations. Darvas et al. [43] studied the competitive adsorption of a neutral amphiphilic polymer and an ionic surfactant at the free water surface by computer simulation methods. Wang et al. [44] explored the adsorption structure of oppositely charged polyelectrolyte and ionic surfactant complexes at the interface using molecular dynamics simulations. Wu et al. [45] reported the effect of the polyacrylamide (PAM) concentration and the hydrolysis degree of HPAM on the adsorption layer of sodium dodecyl sulfate (SDS) at the air–water interface through molecular dynamics simulation methods.

In this work, the water-soluble polymer PVA was added to surfactant solutions, and the foaming ability of surfactant–polymer mixtures was measured through an experiment. Then, the interaction between surfactants and polymers at the air–water interface was simulated through molecular dynamics simulation methods to explain the mechanism responsible for the improved foaming ability following the addition of polymers.

## 2. Materials and Methods

### 2.1. Experimental Details

#### 2.1.1. Materials

Anionic surfactant sodium dodecyl ether sulfate (SDES, purity ≥ 70%) with a formula of CH_3_-(CH_2_)_m_-(OCH_2_CH_2_)_n_-SO_4_Na (m = 11–13, n = 2 or 3) was purchased from Qingdao Yousuo Chemical Technology Co., Ltd. (Qingdao, China). The water-soluble polymer polyvinyl alcohol (PVA1788, purity ≥ 99%) was also provided by the same company. The water used in all experiments was distilled twice. The SDES/PVA mixtures were stirred for 24 h to ensure that the polymer was fully dissolved. All measurements were performed at 25 °C.

#### 2.1.2. Foaming Ability Measurements

**Figure 1:**
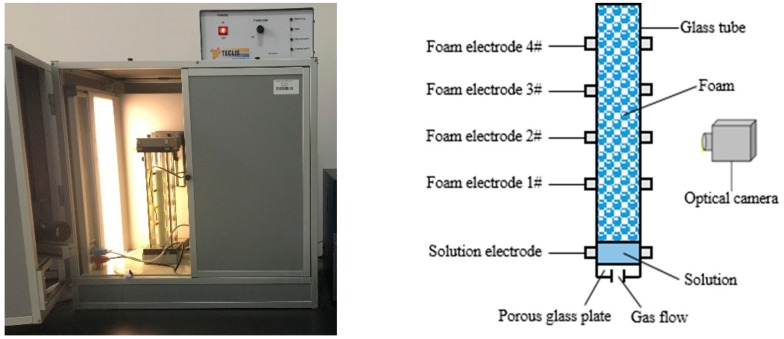
Foamscan analyzer.

A Foamscan analyzer (Teclis Scientific, Lyon, France) was adopted to measure the foaming ability, as shown in Figure 1. Surfactant solutions were injected into a sample cell, which was located at the bottom of a transparent vertical glass tube. Then, gas was dispersed into the solutions through a porous glass plate to generate foam. The foam volume in the glass column was monitored by an optical camera.

In the experiments, there was 40 mL of surfactant solution in the sample cell, and the flow rate of dry air was set at 50 mL/min. The pore diameter of the porous glass plate was 16–40 μm. The experimental temperature was maintained at 25 °C. When the foam volume reached 200 mL, the gas flow was stopped and the foam generation process ended. Foaming ability is characterized by the generation time required to obtain 200 mL of foam—the shorter the generation time, the better the foaming ability is.

### 2.2. Simulation Details

#### 2.2.1. Molecular Structures and Force Field

**Figure 2:**
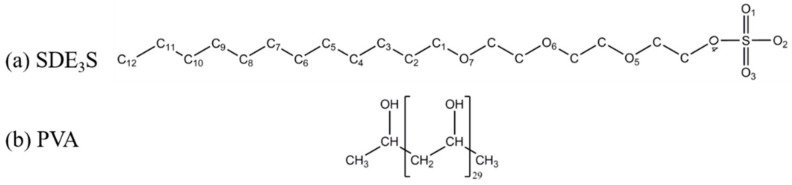
Molecular structures of (**a**) sodium dodecyl ether sulfate containing three oxide groups (SDE_3_S) and (**b**) polyvinyl alcohol (PVA).

In the simulations, surfactant sodium dodecyl ether sulfate containing three polyethylene oxide (EO) groups (SDE_3_S) and a PVA chain consisting of 30 monomer units were adopted. Their molecular structures are shown in Figure 2. There are three types of oxygen atoms in the SDE_3_S hydrophilic head group. The O_1_, O_2_, and O_3_ atoms around the S atom are ionic oxygen atoms. The O_4_ atom between the S atom and the C atom is the ester oxygen atom. The three ether oxygen atoms—O_5_, O_6_, and O_7_—belong to the EO1, EO2, and EO3 groups, respectively.

The GROMACS software package and GROMOS 53A6_OXY+D_ force field were used to perform all of the simulations [46]. The GROMOS 53A6_OXY+D_ force field was developed from the GROMOS 53A6_OXY_ and 53A6 force fields with re-optimized parameters for the oxygen-containing chemical moieties [47,48]. The coordinate files for SDE_3_S and PVA were generated by the Materials Studio 8.0 software, and their topology files were obtained from the topology auto generator ProDRG sever [49], followed by manual adjustments. Detailed force field parameters for SDE_3_S and PVA have been reported in our previous research [50].

#### 2.2.2. Simulation Model

**Figure 3:**
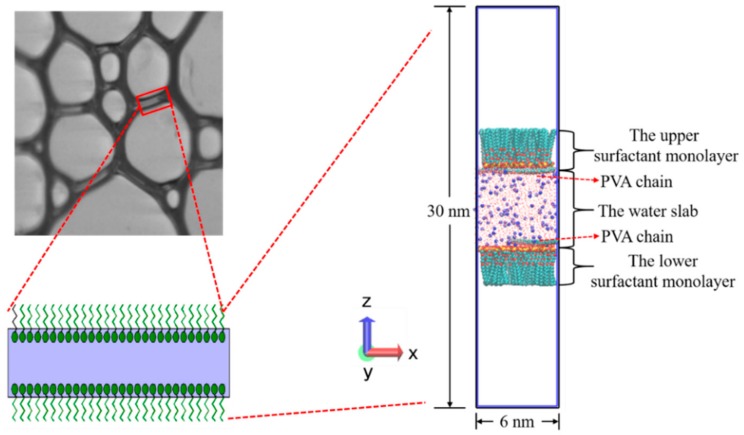
The sandwich model for simulations based on the real liquid film structure in the foam.

Based on the real structure of bubble films, a sandwich model was constructed [51], as shown in Figure 3. A water slab with a thickness of 6 nm bounded by surfactant monolayers was placed at the center of the simulation box with a size of 6 × 6 × 30 nm^3^. Two vacuum regions (each of height ~9 nm) were located below and above the surfactant monolayers, respectively. The two air–water interfaces were perpendicular to the *z*-axis. The SDE_3_S molecules were randomly distributed at the air–water interfaces using the PACKMOL program [52] with the head groups pointing towards the water slab and the tail chains towards the air phase. To simplify the simulation systems, two PVA chains were placed in the water slab within 1 nm from the upper and lower air–water interfaces, respectively.

**Figure 4:**
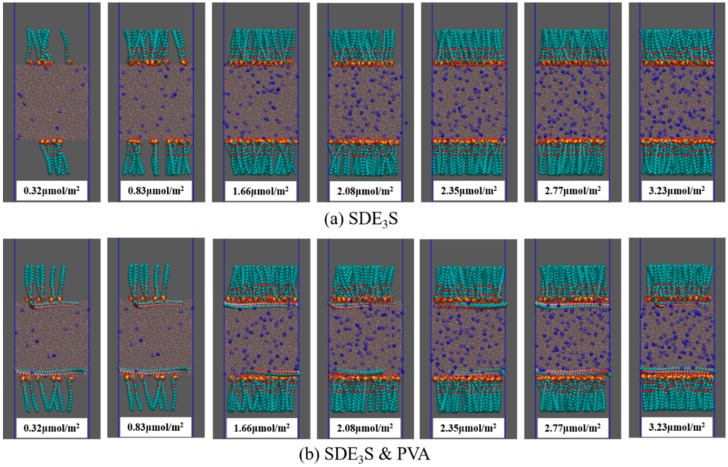
Initial configurations at the beginning of the simulations: (**a**) SDE_3_S systems; (**b**) SDE_3_S/PVA systems.

At saturation coverage, the interfacial area occupied by a single SDE_3_S molecule is approximately 52 Å^2^ [53]. Therefore, each monolayer in the simulation box can hold up to 70 surfactant molecules. The number of SDE_3_S molecules at each interfacial monolayer was set as 7, 18, 36, 45, 51, 60, and 70. The corresponding surfactant interface densities were 0.32, 0.83, 1.66, 2.08, 2.35, 2.77, and 3.23 μmol/m^2^. The initial configurations of SDE_3_S and SDE_3_S/PVA systems at the beginning of the simulations are shown in Figure 4.

#### 2.2.3. Computational Details

Before each simulation, the steepest descent algorithm was used for energy minimization with a tolerance of 1000 kJ mol^−1^ nm^−1^. Then, all the simulations were performed in the canonical ensemble. The temperature was maintained at 298 K using the velocity rescale method with a temperature constant of 0.1 ps [54]. The leapfrog algorithm was used to integrate the equations of motion with a time step of 2 fs [55]. Bond lengths were constrained using the LINCS algorithm [56]. Short-range non-bonded interactions with a cutoff radius of 1.2 nm were employed to determine the Lennard–Jones potential. The long-range electrostatic interactions were computed using the particle mesh Ewald (PME) method with a cutoff radius of 1.2 nm and a grid spacing of 0.16 nm [57]. The simple point charge (SPC) model was used for water molecules [58]. Periodic boundary conditions were applied in all directions. The full simulations were carried out for 60 ns, and the trajectory was saved every 20 ps. The visualization was prepared using VMD 1.9.3 [59].

## 3. Experimental Results and Discussion

**Figure 5:**
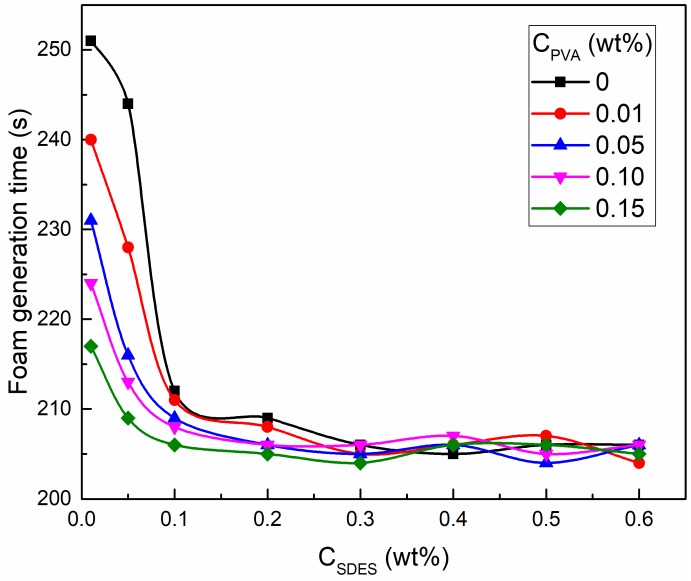
Foam generation time of sodium dodecyl ether sulfate (SDES) and SDES/PVA solutions.

The foam generation time of SDES and SDES/PVA solutions is shown in Figure 5. With an increase in SDES concentration, the foam generation time decreased rapidly and then fluctuated around the stable value. At SDES concentrations of 0.01 and 0.05 wt %, the increase in PVA concentration significantly shortened the foam generation time of SDES solutions. When the SDES concentration was higher than 0.1 wt %, the foam generation time of SDES solutions and SDES/PVA mixed solutions was almost the same. This suggests that the addition of PVA can greatly improve the foaming ability of SDES solutions at low concentrations, which facilitates the concentration reduction of foaming agents.

The reason for these results is explained as follows. During the foam generation process, bubble formation and breakdown occur simultaneously [60,61]. When the bubble formation rate is greater than the breakdown rate, the foam volume increases gradually, but this does not imply that the bubble breakdown phenomenon does not exist. A high bubble rupture rate will lead to a long generation time and low foaming ability. At low SDES concentrations (0.01 and 0.05 wt %), the surfactant interfacial density does not reach saturation, resulting in unstable bubble films and a high breakdown rate, so the foam generation time is relatively long. After adding PVA, the water-soluble polymer can improve the stability of bubble films and reduce the breakdown rate. Thus, as the PVA concentration increases, the foam generation time gradually declines. When the SDES concentration is larger than 0.1 wt %, the surfactant adsorption density at the air–water interface reaches a saturated state, which maintains the stability of bubble films. The presence of PVA has no significant effect on the bubble breakdown rate, so the foam generation time no longer changes.

To prove the improved stability of bubble films by the addition of PVA at low SDES concentrations, calculations were carried out from the perspective of gas supply. In an ideal foam generation process, there would be no bubble breakdown, so the foam volume of 200 mL minus the liquid volume (V_liquid_) in the foam is the ideal gas supply volume (V_ideal gas_). However, in the real foam generation process, bubble rupture exists. Therefore, the difference between the total gas volume (V_total gas_) provided in the real foam generation process and the V_ideal gas_ is the volume of gas (ΔV) escaped from the bursting bubbles. The larger the ΔV value is, the worse the stability of bubble films is, and the more serious the bubble rupture phenomenon is. The V_total gas_, V_ideal gas_, and ΔV values of SDES/PVA solutions at SDES concentrations of 0.01 and 0.05 wt % are listed in Table 1 and Table 2, respectively. With an increase in the PVA concentration, ΔV values exhibited a downward trend, which indicates that the volume of gas released from bursting bubbles gradually decreased. This demonstrates that the addition of PVA helps to improve the stability of bubble films.

## 4. Simulation Results and Discussion

**Figure 6:**
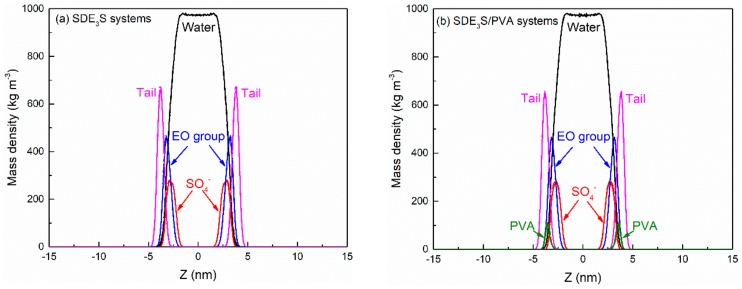
Mass density profiles of (**a**) SDE_3_S systems and (**b**) SDE_3_S/PVA systems over both 30–40 ns (solid lines) and 40–50 ns (dashed lines) at the SDE_3_S adsorption density of 3.23 μmol/m^2^.

The equilibration of the simulation systems can be judged by monitoring the mass density profiles [62]. At an SDE_3_S adsorption density of 3.23 μmol/m^2^, the mass density profiles of SDE_3_S and SDE_3_S/PVA systems over 30–40 ns and 40–50 ns were as shown in Figure 6. It can be seen that the mass density profiles converge in the two intervals, which demonstrates that the simulation systems reached equilibration after 30 ns. If the simulation system with the largest number of surfactants has reached equilibration, a shorter time is required for other simulation systems to achieve equilibrium. Therefore, the trajectory data of the last 10 ns were used for analysis in all simulations.

**Figure 7:**
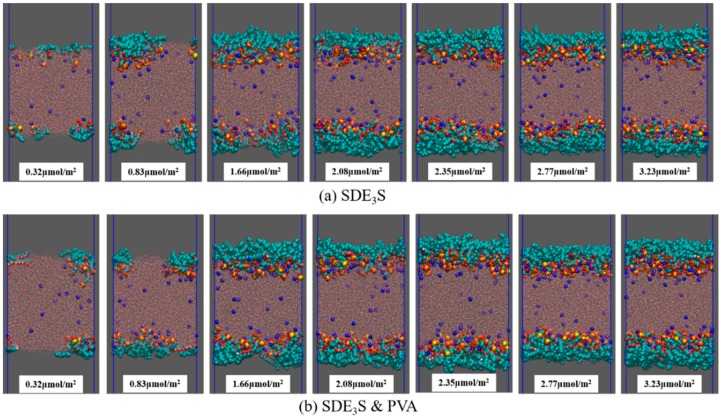
Final configurations at the end of simulations: (**a**) SDE_3_S systems; (**b**) SDE_3_S/PVA systems.

The final configurations of the SDE_3_S and SDE_3_S/PVA systems at the end of the simulations are presented in Figure 7. It can be seen that the surfactant head groups extended into the bulk water, and the tail chains were distributed in the air phase, which is consistent with the adsorption state of surfactants at the real air–water interface. In addition, the two PVA chains migrated from the bulk water into the surfactant adsorption monolayers, suggesting that an interaction between SDE_3_S molecules and PVA chains occurred.

### 4.1. Density Profiles

**Figure 8:**
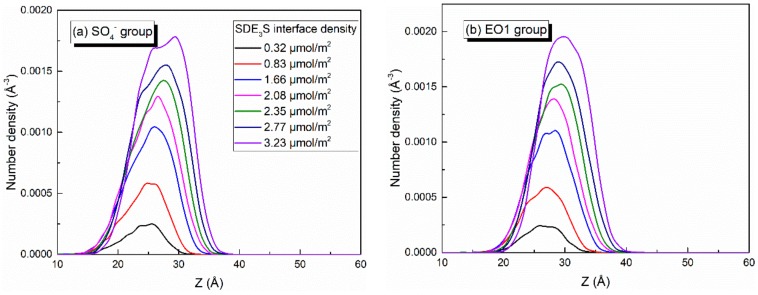

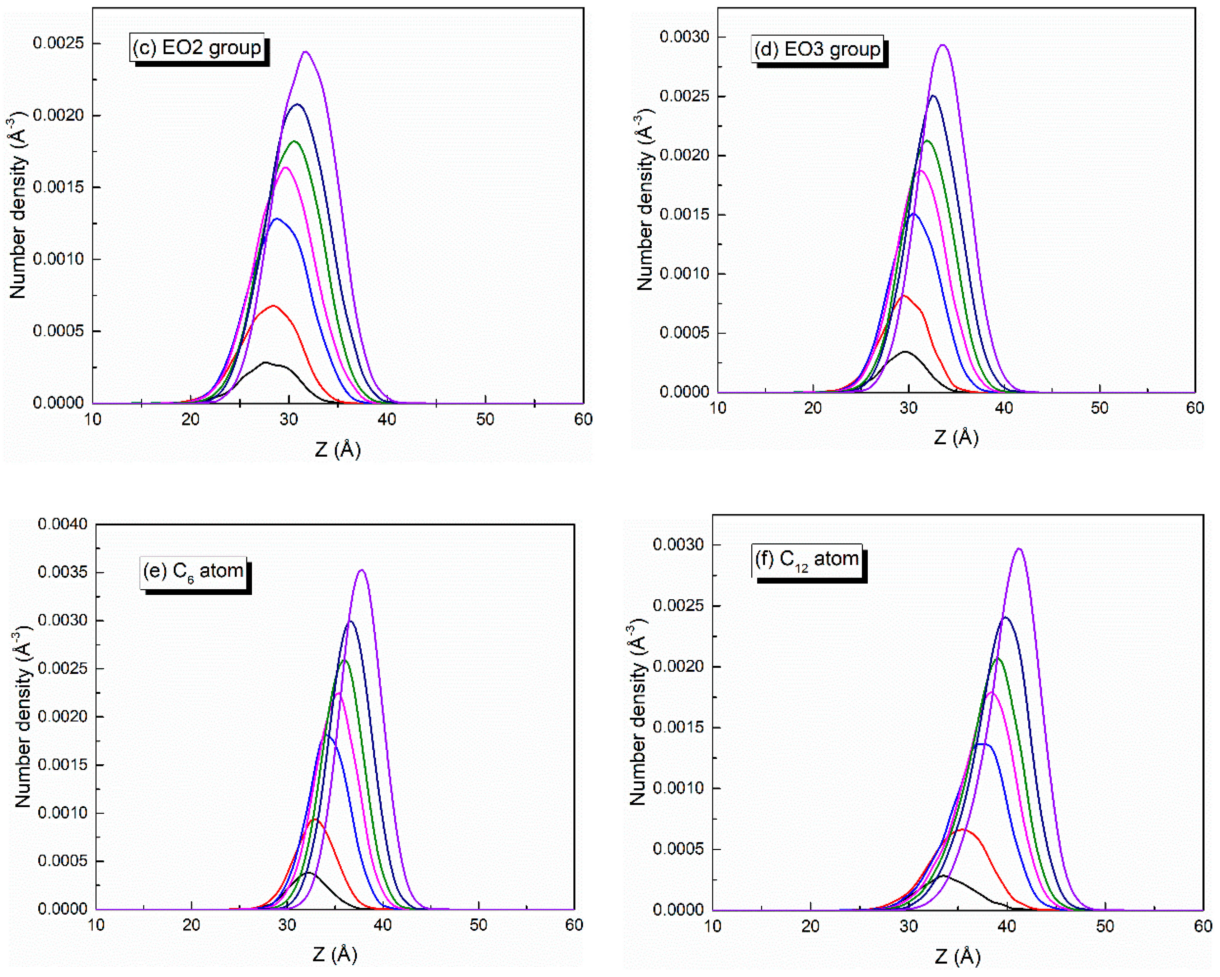
Number density profiles of (**a**) SO_4_^−^, (**b**) EO1, (**c**) EO2, (**d**) EO3, (**e**) C_6_, and (**f**) C_12_ in SDE_3_S systems.

**Figure 9:**
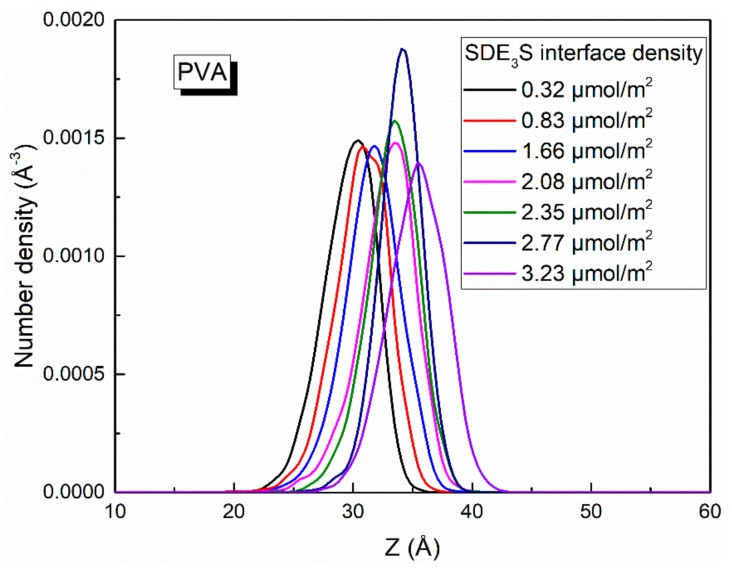
Number density profiles of PVA in SDE_3_S/PVA systems.

Since the bubble film structure is symmetric about the center of the water slab, the center position of the water slab was taken as the zero point of the *z*-axis. When calculating the number density profiles of different atoms or groups along the z direction, the data from the upper and lower parts were averaged and plotted. At different adsorption densities of SDE_3_S at the air–water interface, the number density profiles of SO_4_^−^, EO1, EO2, EO3, C_6_, and C_12_ atoms or groups were as shown in Figure 8. As the SDE_3_S interface density increased, the distribution range of the SO_4_^−^, EO1, EO2, and EO3 groups widened, which could attract more water molecules to migrate around them, resulting in an increase in the thickness of the interface layer. Moreover, with the increase of the surfactant interface density, the peak positions of the number density curves of SO_4_^−^, EO1, EO2, EO3, C_6_, and C_12_ atoms or groups increased by 4–5 Å. In the SDE_3_S/PVA mixed systems, Figure 9 reveals that the PVA chains also migrated along the *z*-axis toward the air phase by 5 Å with the increase of the SDE_3_S adsorption density. The same migration distance suggests there were stable interactions between SDE_3_S molecules and PVA chains.

### 4.2. Surfactant Orientation at the Air–Water Interface

**Figure 10:**
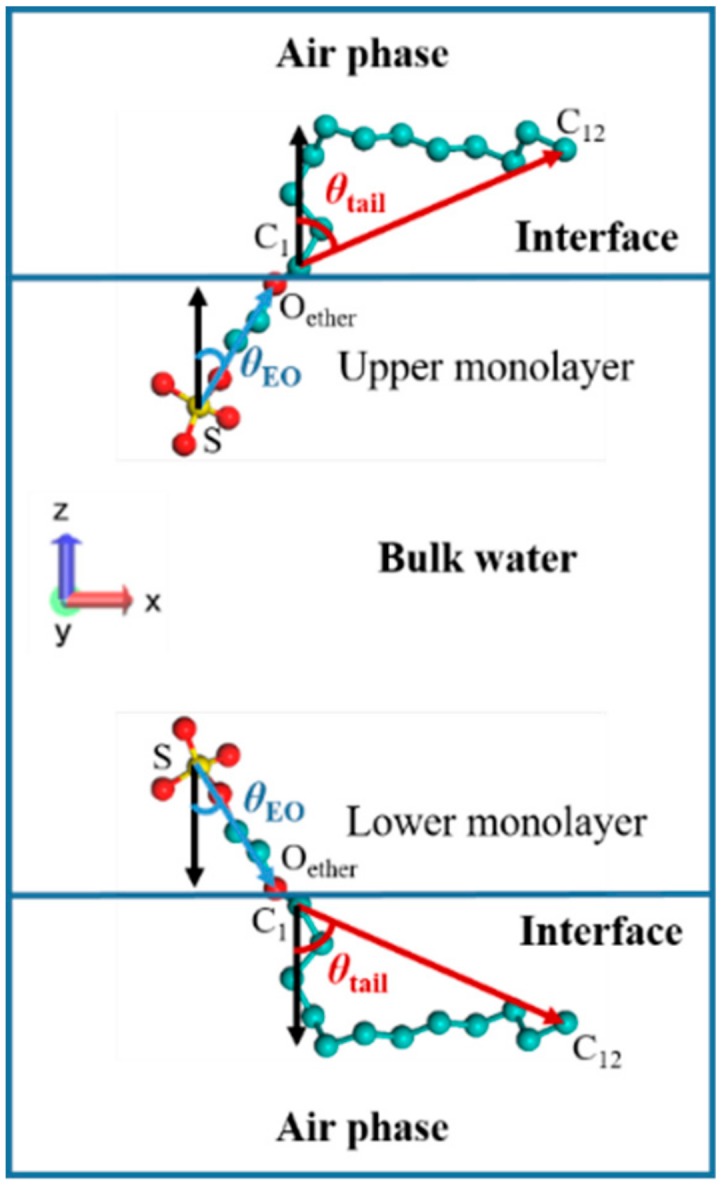
Definitions of the tilt angles of the polyethylene oxide (EO) group and tail chain in SDE_3_S surfactant at the air–water interface.

The definitions of the tilt angles of the EO group and tail chain at the air–water interface are illustrated in Figure 10. The vector pointing along the hydrophilic head group from the S atom to the ether oxygen atom (O_ether_) was defined as the EO group vector, and the vector pointing along the hydrophobic tail group from the C_1_ atom to the C_12_ atom was defined as the tail vector. In the upper surfactant monolayer, the angles between the two vectors and the negative *z*-axis direction were defined as θ_EO_ and θ_tail_, respectively, while in the lower surfactant monolayer, the angles of the EO group vector and the tail vector with a positive *z*-axis direction were defined as θ_EO_ and θ_tail_, respectively. As can be found from Figure 10, the range of θ_EO_ and θ_tail_ was 0°–90°. When θ_EO_ or θ_tail_ equaled 0°, this implies that the EO group or tail chain was perpendicular to the air–water interface, whereas when θ_EO_ or θ_tail_ equaled 90°, the EO group or tail chain was parallel to the interface. Since there are three EO groups in the structure of SDE_3_S molecule, their tilt angles were named θ_EO1_, θ_EO2_, and θ_EO3_, respectively.

**Figure 11:**
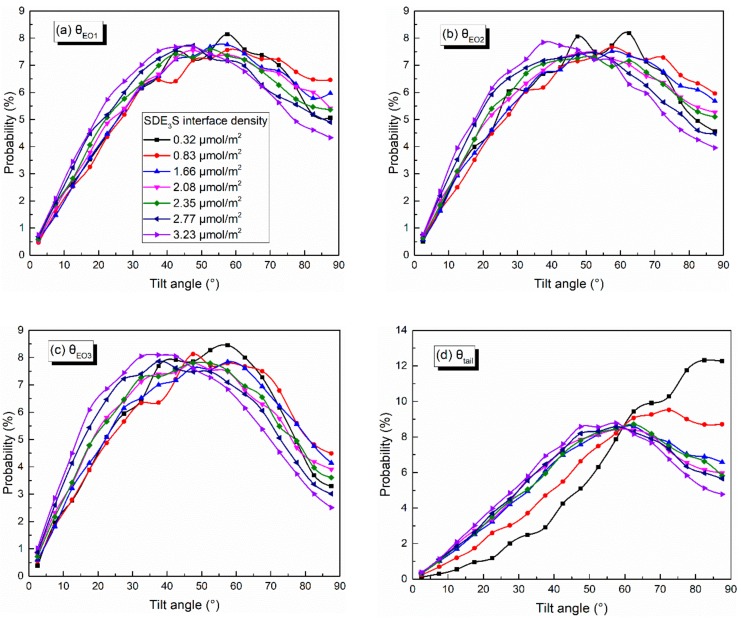
Tilt angles of (**a**) θ_EO1_, **(b**) θ_EO2_, (**c**) θ_EO3_, and (**d**) θ_tail_ in SDE_3_S systems.

The distributions of the tilt angles of θ_EO1_, θ_EO2_, θ_EO3_, and θ_tail_ in the SDE_3_S simulation systems are shown in Figure 11. With the increase in the surfactant interface density, the percentages of θ_EO1_, θ_EO2_, and θ_EO3_ values lower than 47.5° increased gradually, while the proportion of those greater than 47.5° decreased significantly, especially for θ_EO1_, θ_EO2_, and θ_EO3_ with an angle of 87.5°. In addition, when the interface density was 0.32 μmol/m^2^, the angle of θ_EO1_ corresponding to the largest proportion was 57.5°. At the interface density of 3.23 μmol/m^2^, the angle of θ_EO1_ corresponding to the largest percentage decreased to 47.5°. As the surfactant adsorption density increased, the angle of θ_EO2_ at the highest ratio decreased from 62.5° to 37.5°, and the angle of θ_EO3_ at the highest proportion decreased from 57.5° to 37.5°. This indicates that the hydrophilic head groups became more perpendicular to the air–water interface with the increase in the surfactant adsorption density. Figure 11d shows that, when increasing the surfactant interface density, the ratio of θ_tail_ larger than 60° declined, and the percentage of θ_tail_ smaller than 60° increased. The angle of θ_tail_ corresponding to the highest proportion reduced from 82.5° to 57.5°. The ratio of θ_tail_ equaling 87.5° declined most obviously, from 12.26% to 4.78%. This suggests that an increase in the surfactant adsorption density makes the tail chain vertical to the interface, rather than lying flat along the interface.

**Figure 12:**
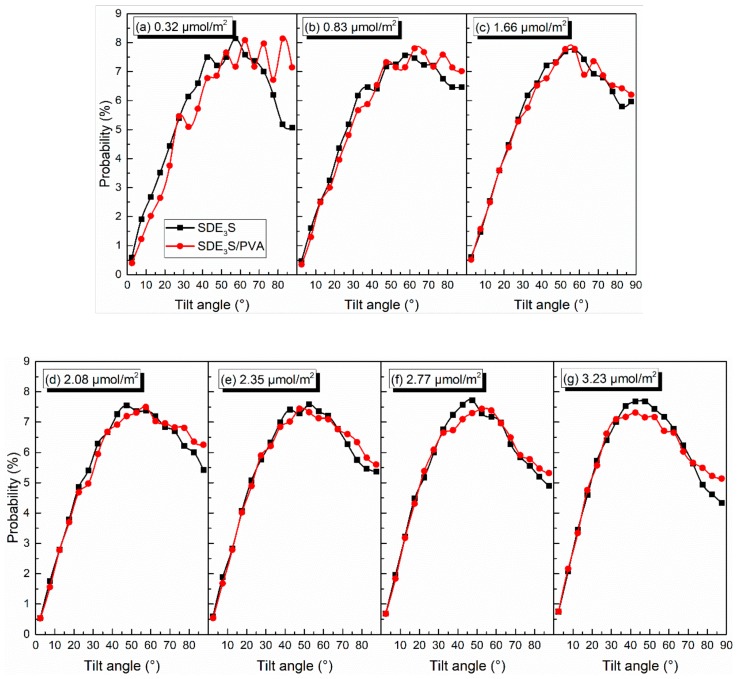
Comparison of tilt angles of θ_EO1_ in SDE_3_S and SDE_3_S/PVA systems: (**a**) 0.32 μmol/m^2^; (**b**) 0.83 μmol/m^2^; (**c**) 1.66 μmol/m^2^; (**d**) 2.08 μmol/m^2^; (**e**) 2.35 μmol/m^2^; (**f**) 2.77 μmol/m^2^; (**g**) 3.23 μmol/m^2^.

**Figure 13:**
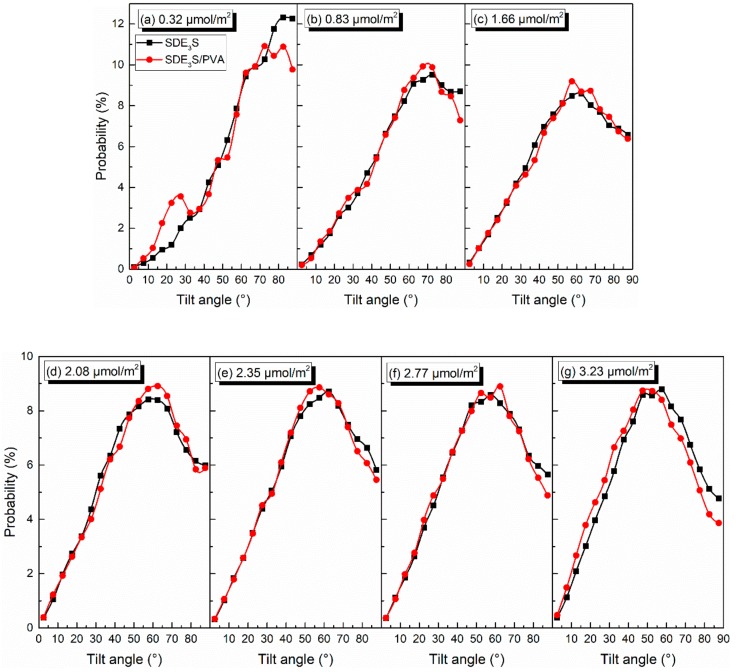
Comparison of tilt angles of θ_tail_ in SDE_3_S and SDE_3_S/PVA systems: (**a**) 0.32 μmol/m^2^; (**b**) 0.83 μmol/m^2^; (**c**) 1.66 μmol/m^2^; (**d**) 2.08 μmol/m^2^; (**e**) 2.35 μmol/m^2^; (**f**) 2.77 μmol/m^2^; (**g**) 3.23 μmol/m^2^.

Taking θ_EO1_ and θ_tail_ as examples, the effect of PVA on the tilt angles of the surfactant hydrophilic head group and tail chain was investigated. The comparison of θ_EO1_ and θ_tail_ in SDE_3_S and SDE_3_S/PVA systems is shown in Figure 12 and Figure 13, respectively. It can be seen from Figure 12 that the proportion of θ_EO1_ with large angle values in the SDE_3_S/PVA systems was higher than that in the SDE_3_S systems, indicating that the hydrophilic head groups of SDE_3_S molecules were more parallel to the air–water interface after adding PVA. This favors an increase in the coverage area of the hydrophilic groups at the interface and strengthens the hydration of surfactants, thereby enhancing the stability of bubble films. In Figure 13, the ratio of θ_tail_ with large values in the SDE_3_S/PVA systems is shown to be lower than that in the SDE_3_S systems. The presence of PVA makes the surfactant tail chain tend to be vertical to the interface, indicating that the tail chain is further away from the bulk water. Because the tail chain is hydrophobic, it can promote binding between water molecules and surfactant hydrophilic head groups indirectly, which is favorable for improving the stability of bubble films.

### 4.3. Hydration Structures of Surfactant Oxygen Atoms

**Figure 14:**
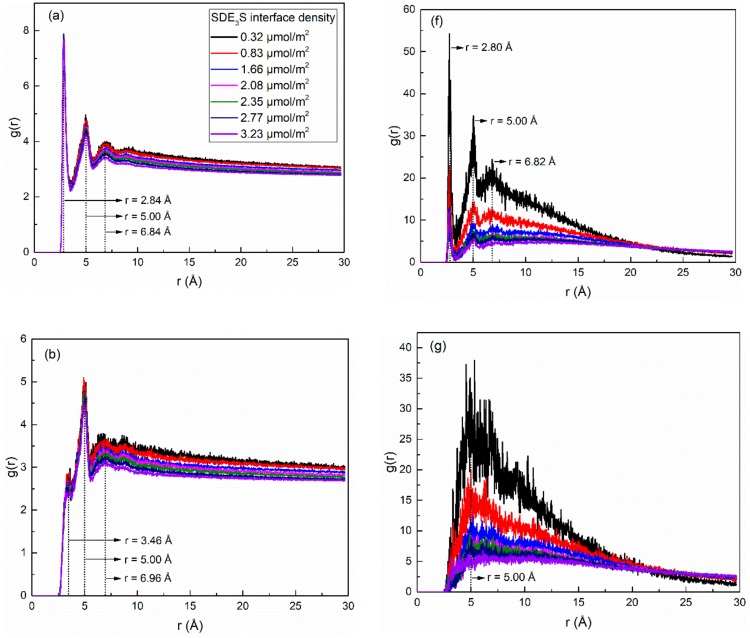

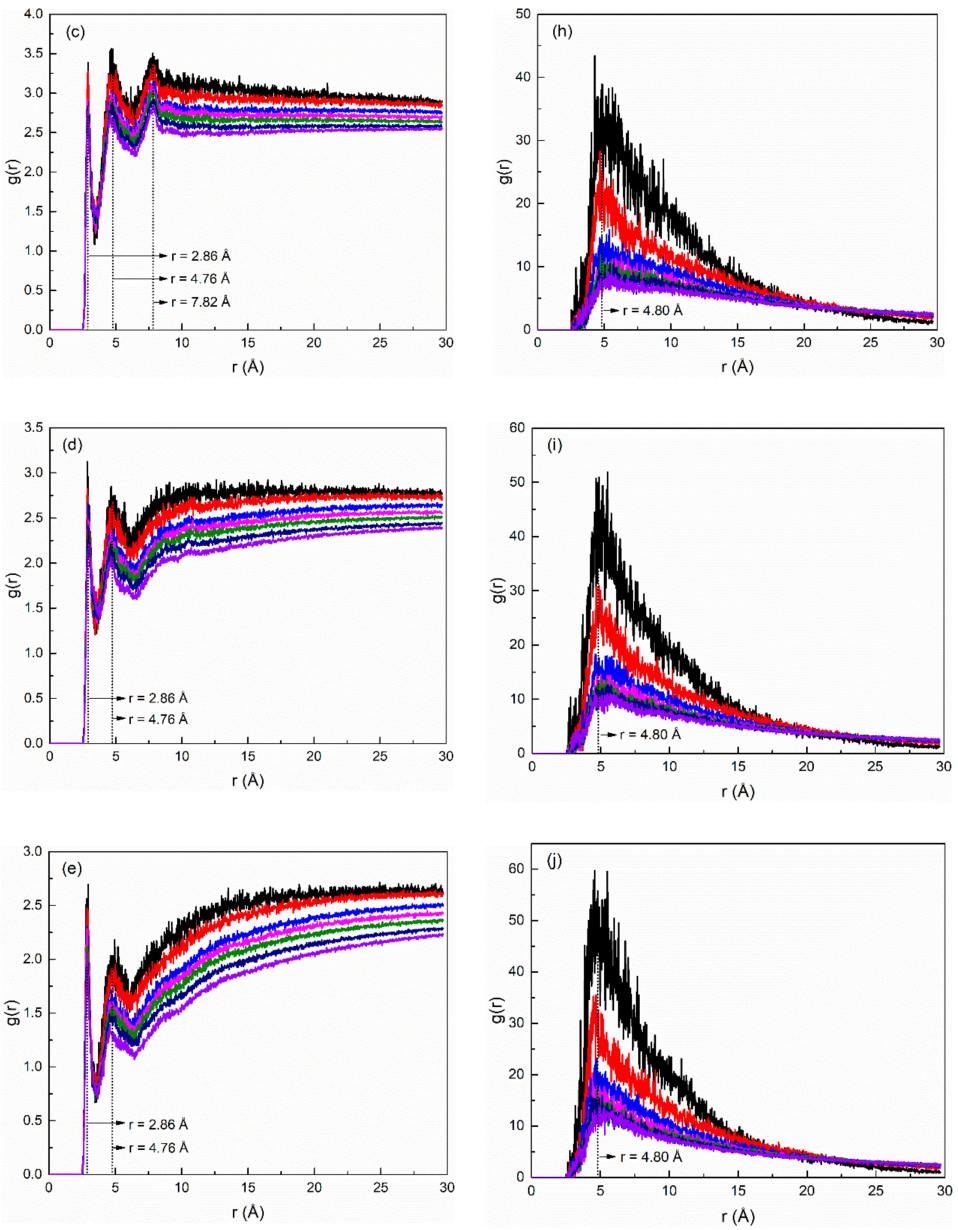
Radial distribution functions of water atoms (represented by their oxygen atoms) and PVA (represented by its oxygen atoms) around different oxygen atoms of surfactant in SDE_3_S/PVA systems: (**a**) O_1-3_-O_W_, (**b**) O_4_-O_W_, (**c**) O_5_-O_W_, (**d**) O_6_-O_W_, (**e**) O_7_-O_W_, (**f**) O_1-3_-O_PVA_, (**g**) O_4_-O_PVA_, (**h**) O_5_-O_PVA_, (**i**) O_6_-O_PVA_, (**j**) O_7_-O_PVA_.

In order to study the structure of hydration shells of the surfactant head group in the SDE_3_S/PVA systems, the radial distribution functions (RDFs) of water oxygens around the three types of oxygen atoms in the surfactant head group were calculated and are displayed in Figure 14a–e. In Figure 14a, the RDF curves of the ionic oxygen atoms exhibit three peaks corresponding to three hydrated water shells at positions of 2.84, 5.00, and 6.84 Å, respectively. It can be seen from Figure 14b that the ester oxygen atom O_4_ also formed three hydration shells. The position of the second hydration shell was the same as that of the ionic oxygen atoms, but the first and third hydration layers were located at 3.46 and 6.96 Å, respectively. As shown in Figure 14c, the ether oxygen atom O_5_ in the EO1 group formed three hydration shells at 2.86, 4.76, and 7.82 Å, while the ether oxygen atoms O_6_ and O_7_ in the EO2 and EO3 groups only formed two hydration layers at 2.86 and 4.76 Å, as shown in Figure 14d,e.

The RDFs of PVA oxygen atoms around the three types of surfactant oxygen atoms are given in Figure 14f–j. In Figure 14f, the RDF curves of O_1-3_-O_PVA_ show three peaks (r = 2.80, 5.00, and 6.82 Å) at almost the same positions of the three hydration shells of the ionic oxygen atoms, indicating that the water-soluble polymer PVA was distributed in the hydration layers of SDE_3_S ionic oxygen atoms. Figure 14g shows only one peak located at 5.00 Å in the O_4_-O_PVA_ RDF curves, which is the same as the position of the second hydration shell of the ester oxygen atom. It can be seen from Figure 14h–j that the peak position in the O_5_-O_PVA_, O_6_-O_PVA_, and O_7_-O_PVA_ RDF curves was at 4.80 Å, which is close to the position of the second hydration shell of the ether oxygen atoms. Due to the strong hydrophilic ability of the hydroxyl groups, the presence of PVA in the hydration shells of the different oxygen atoms can enhance the hydration ability of the surfactant head group, thereby improving the stability of bubble films in aqueous foam.

### 4.4. Hydrogen Bonds

**Figure 15:**
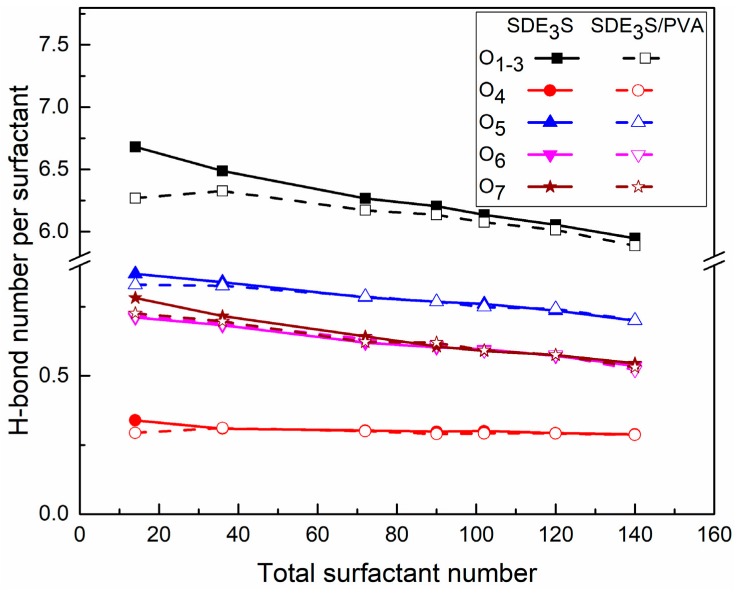
Hydrogen bond numbers per surfactant between different oxygen atoms in surfactant and water molecules in SDE_3_S and SDE_3_S/PVA systems.

In the SDE_3_S and SDE_3_S/PVA simulation systems, the number of hydrogen bonds (H-bonds) per surfactant formed between different types of head-group oxygen atoms and water molecules is shown in Figure 15. The ability of different oxygen atoms to form H-bonds was found to differ, decreasing in the order of ionic oxygen, ether oxygen, and ester oxygen. The number of hydrogen bonds formed by ionic oxygen atoms in each surfactant was the largest (about 5.8 to 6.7), followed by the O_5_ atom in the EO1 group (0.7 to 0.87). The range of H-bond numbers formed by the O_6_ and O_7_ atoms in the EO2 and EO3 groups was from 0.52 to 0.78, slightly lower than that formed by the O_5_ atom. The ester oxygen atom O_4_ was shown to have the weakest hydrogen bonding ability, and only formed about 0.3 hydrogen bonds. The above results suggest that the hydration ability of the SDE_3_S head group mainly depends on the ionic oxygen atoms, but the presence of EO groups can further increase the number of hydrogen bonds.

**Figure 16:**
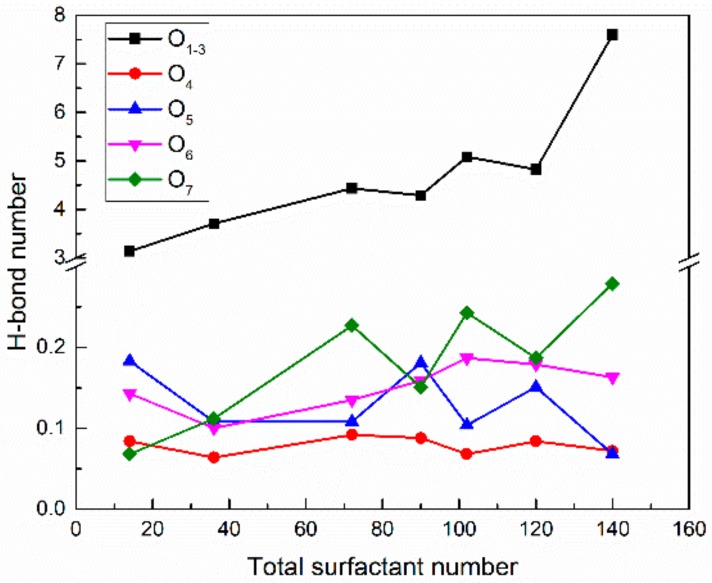
Hydrogen bond numbers between different oxygen atoms and PVA in SDE_3_S/PVA systems.

**Figure 17:**
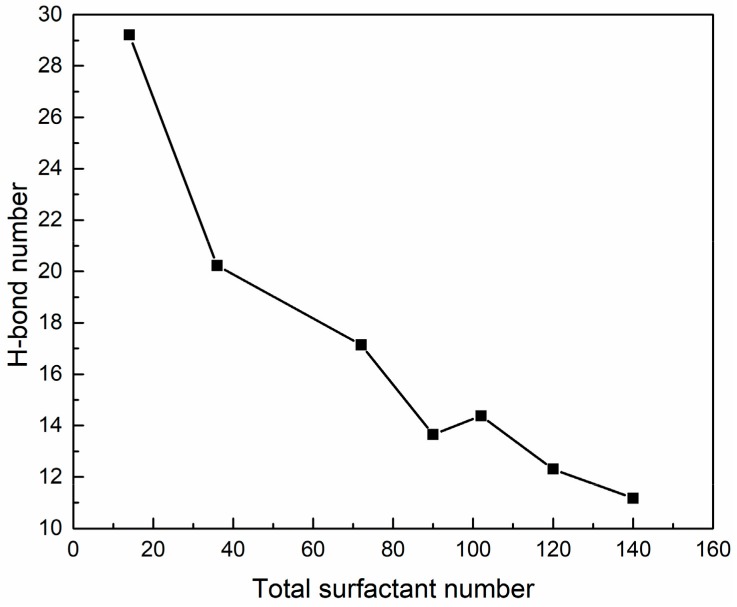
Hydrogen bond numbers between PVA and water molecules in SDE_3_S/PVA systems.

Figure 15 also shows that as the surfactant number increased, the H-bond number per surfactant formed by different oxygen atoms decreased gradually. One explanation for this is that the crowded air–water interface made the hydrophilic head groups migrate away from the bulk water. Moreover, the addition of PVA did not affect the H-bond numbers of the ether and ester oxygen atoms but slightly reduced the H-bond number formed by the ionic oxygen atoms. This is because the interaction between ionic oxygen atoms and PVA was the strongest. After adding PVA, some ionic oxygen atoms formed hydrogen bonds with the hydroxyl groups of PVA, as shown in Figure 16, thus reducing the number of available ionic oxygen atoms that form H-bonds with water molecules. Although the H-bond number between ionic oxygen atoms and water molecules in SDE_3_S/PVA systems is slightly less than that in SDE_3_S systems, a large number of hydroxyl groups contained in PVA can form many hydrogen bonds with water molecules, as shown in Figure 17. The presence of PVA actually increased the total number of oxygen-containing groups at the air–water interface, which could improve the hydration ability of the surfactant adsorption monolayers.

The H-bond numbers between surfactant oxygen atoms and PVA in the SDE_3_S/PVA systems are given in Figure 16. It can be seen that the ionic oxygen atoms formed 3.1 to 7.6 H-bonds with PVA, followed by the ether oxygen atoms (about 0.1 to 0.28) and ester oxygen (about 0.06 to 0.09). Figure 17 shows that as the surfactant number increased, the H-bond numbers formed between PVA and water molecules decreased from 29.20 to 11.18. This is because the crowded interface gradually pushed PVA away from the bulk water, thus reducing the chance of PVA contacting water molecules. The above results indicate that hydrogen bonds can be formed among surfactant hydrophilic head groups, PVA, and water molecules. In this way, a widely connected, three-dimensional H-bond network is shaped in the adsorption layer at the air–water interface. Therefore, the stability of bubble films is enhanced by the addition of PVA. When the surfactant number is the smallest, the H-bond number between PVA and water molecules is the largest. Therefore, the improvement of bubble film stability is the most obvious, which reasonably suggests that the foaming ability of SDES solutions was significantly improved by the addition of PVA at low concentrations in the experiment.

## 5. Conclusions

This study investigated the effect of water-soluble polymer PVA on the foaming ability of surfactant SDES solutions through an experiment and then revealed the interaction between SDE_3_S and PVA at the air–water interface through molecular dynamics simulations. This was used to explain how the polymer improves the stability of bubble films and the foaming ability of surfactant solutions. The key conclusions are summarized as follows.

At SDES concentrations lower than 0.1 wt %, the addition of PVA significantly shortens the foam generation time of SDES solutions. When the SDES concentration is higher than 0.1 wt %, the foam generation time is almost the same for SDES solutions and SDES/PVA mixed solutions. This suggests that the presence of PVA can greatly improve the foaming ability of SDES solutions at low concentrations, which is helpful for reducing the concentration of foaming agents. The reason for this is that at low SDES concentrations, the surfactant interfacial density does not reach saturation, resulting in unstable bubble films and a high breakdown rate. After adding PVA, the water-soluble polymer can enhance the stability of bubble films and reduce the breakdown rate of bubbles, thereby shortening the foam generation time and improving the foaming ability.

The simulation results show that the hydrophilic head groups of SDE_3_S molecules are more parallel to the air–water interface after adding PVA. This favors an increase in the coverage area of hydrophilic groups at the interface and strengthens the hydration of surfactants. In addition, the presence of PVA makes the surfactant tail chain tend to be vertical to the interface, which means the tail chain is further away from the bulk water. Because the tail chain is hydrophobic, it can indirectly promote binding between water molecules and surfactant hydrophilic groups. Radial distribution functions reveal that PVA appears in the hydration shells of the different oxygen atoms in the SDE_3_S head group. Since a large number of hydroxyl groups contained in PVA have a strong hydrophilic ability, this helps to enhance the hydration of the surfactant head groups. Hydrogen bonds can be formed among surfactant hydrophilic head groups, PVA, and water molecules. In this way, a widely connected, three-dimensional H-bond network is shaped in the adsorption layer at the air–water interface.

The above simulation analysis results indicate that the addition of PVA can enhance the stability of bubble films of SDES solutions. When the surfactant interface density is the lowest, the improvement in bubble film stability is the most obvious, which reasonably suggests that the foaming ability of SDES solutions was significantly improved by the addition of PVA at low concentrations in the experiment. Therefore, water-soluble polymers can be used to improve the foaming ability of surfactant solutions, which helps to reduce the added concentration of foaming agents in foam extinguishing technology and foam dust suppression technology.

## Figures and Tables

**Table 1:** The values of the volume of gas (ΔV), total gas volume (V_total gas_) and ideal gas supply volume (V_ideal gas_) at an SDES concentration of 0.01 wt %.

SDES Concentration 0.01 wt %	PVA Concentration				
	0 wt %	0.01 wt %	0.05 wt %	0.1 wt %	0.15 wt %
V_total gas_ (mL)	209.17	200	192.5	186.67	180.83
V_ideal gas_ (mL)	190.92	187.29	184.4	181.37	178.3
ΔV (mL)	18.25	12.71	8.1	5.3	2.53

**Table 2:** The values of ΔV, V_total gas_, and V_ideal gas_ at an SDES concentration of 0.05 wt %.

SDES Concentration 0.05 wt %	PVA Concentration				
	0 wt %	0.01 wt %	0.05 wt %	0.1 wt %	0.15 wt %
V_total gas_ (mL)	203.33	190	180	177.5	174.17
V_ideal gas_ (mL)	184.84	178.18	175.34	173.8	171.72
ΔV (mL)	18.49	11.82	4.66	3.7	2.45
